# Association between Brain-Derived Neurotrophic Factor (BDNF) Levels in 2^nd^ Trimester Amniotic Fluid and Fetal Development

**DOI:** 10.1155/2018/8476217

**Published:** 2018-12-05

**Authors:** Nikolaos Antonakopoulos, Zoe Iliodromiti, George Mastorakos, Christos Iavazzo, Georgios Valsamakis, Nikolaos Salakos, Aris Papageorghiou, Alexandra Margeli, Sophia Kalantaridou, George Creatsas, Efthymios Deligeoroglou, Nikolaos Vrachnis

**Affiliations:** ^1^Second Department of Obstetrics and Gynecology, National and Kapodistrian University of Athens Medical School, Aretaieio Hospital, Athens, Greece; ^2^Gynecological Oncology Department, Metaxa Cancer Hospital, Piraeus, Greece; ^3^Department of Endocrinology and Metabolic Disorders, University of Thessaly Medical School, Larissa University Hospital, Larissa, Greece; ^4^St George's University of London Medical School and St George's University Hospitals NHS Foundation Trust, London, UK; ^5^Nuffield Department of Obstetrics and Gynecology, University of Oxford, Oxford, UK; ^6^Department of Clinical Biochemistry, “Aghia Sophia” Children's Hospital, Athens, Greece

## Abstract

The development of the fetal nervous system mirrors general fetal development, comprising a combination of genetic resources and effects of the intrauterine environment. Our aim was to assess the 2^nd^ trimester amniotic fluid levels of brain-derived neurotrophic factor (BDNF) and to investigate its association with fetal growth. In accordance with our study design, samples of amniotic fluid were collected from women who had undergone amniocentesis early in the 2^nd^ trimester. All pregnancies were followed up until delivery and fetal growth patterns and birth weights were recorded, following which pregnancies were divided into three groups based on fetal weight: (1) AGA (appropriate for gestational age), (2) SGA (small for gestational age), and (3) LGA (large for gestational age). We focused on these three groups representing a reflection of the intrauterine growth spectrum. Our results revealed the presence of notably higher BDNF levels in the amniotic fluid of impaired growth fetuses by comparison with those of normal growth. Both SGA and macrosomic fetuses are characterized by notably higher amniotic fluid levels of BDNF (mean values of 36,300 pg/ml and 35,700 pg/ml, respectively) compared to normal-growth fetuses (mean value of 32,700 pg/ml). Though apparently small, this difference is, nevertheless, statistically significant (*p* value < 0.05) in SGA fetuses in the extremes of the distribution, i.e., below the 3rd centile. In conclusion, there is clear evidence that severe impairment of fetal growth induces the increased production of fetal brain growth factor as an adaptive mechanism in reaction to a hostile intrauterine environment, thereby accelerating fetal brain development and maturation.

## 1. Introduction

Impaired or excessive fetal growth leads to increased rates of perinatal morbidity and mortality [1]. A small for gestational age (SGA) fetus usually refers to a fetus with an estimated fetal weight (EFW) less than the 10^th^ centile, while a severe SGA is a fetus of estimated fetal weight less than the 3rd centile, with the risk of negative pregnancy outcome increasing as we move from small to severe small for gestational age fetuses [2, 3]. Recent data support the additional use of customised birth weight centiles for maternal characteristics, including maternal parity, ethnic origin, height, and weight [4, 5]. Certainly, early antenatal recognition of gestational characteristics allows for closer follow-up and targeted interventions, which are likely to improve outcomes [6].

Fetal growth restriction (FGR) is not identical with SGA, as FGR denotes a pathological constraint of the genetic growth potential. FGR refers to a fetus whose estimated weight is below the 10^th^ centile for gestational age and who additionally displays signs of chronic malnutrition and hypoxia. The likelihood of growth restriction increases dramatically in severe SGA fetuses. While all structurally normal SGA fetuses are at higher risk of perinatal mortality and morbidity, the majority of adverse outcomes occur among the FGR group [7]. Importantly, however, there is a greater incidence of negative long-term neurodevelopmental, cardiovascular, and endocrinological outcomes among both SGA and FGR fetuses [8]. In any case, a very low EFW (<3rd centile) points to adverse perinatal outcomes regardless of the presence of other indices such as abnormal Doppler findings [9, 10]. Therefore, all otherwise normal fetuses with an EFW below the 3^rd^ centile, as well as those fetuses with an EFW below the 10^th^ centile who manifest signs of fetal compromise, should be considered as FGR, a process mainly caused by chronic placental insufficiency, and need to be closely monitored due to the high risk for an adverse outcome. Moreover, macrosomic or large for gestational age (LGA) fetuses are those with a weight above the 90^th^ centile for gestational age. Though late-gestation fetal growth lowers the risk of perinatal mortality, fetal macrosomia can cause labor complications that may raise the risk of perinatal death [1, 11].

Compromised general fetal development will also affect prenatal central nervous system development, the forming of the most complex structure within the human being. Among these developmental risks is, as mentioned above, chronic placental insufficiency, which can lead to long-lasting deficits in neuronal connectivity and function. Both the severity and the timing of these prenatal insults will determine which regions of the brain are affected and how serious will be the damage [12].

The pathophysiological mechanisms behind impaired or excessive fetal growth have not as yet been entirely elucidated. Despite numerous research efforts, no molecular prognostic marker has to date been identified. We studied a neurotrophic factor which protects fetal nervous system development, differentiation, and metabolism, i.e., brain-derived neurotrophic factor (BDNF). BDNF is a member of the neurotrophic growth factor family: it is 52% identical to nerve growth factor (NGF), the neuropeptide that is mainly involved in the regulation of proliferation, growth, maintenance, and survival of a number of target neurons [13, 14]. Among the cells expressing the BDNF molecule are fibroblasts [15], astrocytes [16], neurons of various localizations and phenotypes [16–18], megakaryocyte-platelets [19], Schwann cells [20], and possibly smooth muscle cells [21]. Two distinct receptors of BDNF have been identified, low-affinity 75 kDa LNGFR (low-affinity nerve growth factor receptor) and high-affinity 145 kDa TrkB (tropomyosin receptor kinase-B) [22]. These receptors, after binding BDNF, have important roles to play, namely, implication in growth, differentiation and survival, reverse transportation in neurons, induction of Schwann cell migration, synaptogenesis, and lymphopoiesis [20, 23–26]. To date, while human adult, neonatal, and animal fetal studies involving measurements of BDNF and correlation of these with several aspects of neural growth and function have been carried out, there has been no comparable human fetal research.

The purpose of this study is the detection and quantification of BDNF in the amniotic fluid of 2^nd^ trimester pregnancies. Furthermore, we aim to draw attention to potential correlations between BDNF levels in amniotic fluid and impaired fetal growth as a means of gaining greater insight into the mechanisms underlying fetal growth restriction and macrosomia, which have been linked to maternal, fetal, and neonatal adverse outcomes. It is thus hoped that the present study may reveal a possible prognostic role of this factor as measured in the 2^nd^ trimester and in pregnancy outcome as revealed in the 3^rd^ trimester.

## 2. Materials and Methods

Amniotic fluid samples were collected from women who had undergone amniocentesis early in the 2^nd^ trimester of gestation (15–22 weeks) based on various indications, such as advanced maternal age, increased nuchal translucency, previous history of birth defects, or detection of an anomaly in the ultrasound examination of the first or 2^nd^ trimester. Immediately after amniocentesis, the amniotic fluid samples were centrifuged and stored in polypropylene tubes at −80°C. Excluded from the study were twin pregnancies and pregnancies with fetuses of abnormal karyotype or severe congenital malformations. All pregnancies were followed up until delivery. Fetal growth patterns and birth weights were recorded and subsequently divided into three groups: SGA (small for gestational age), AGA (appropriate for gestational age), and LGA (large for gestational age). A gestation-related weight computer program was used to allocate the centile of each neonate at delivery [27]. Our study sample was composed of 31 SGA fetuses and 18 LGA fetuses matched for gestational age, sex, maternal height, and weight and compared with 31 AGA fetuses composing the control group. The corresponding amniotic fluid samples were then withdrawn from our amniotic fluid sample bank, and BDNF was measured in order to compare its levels between normal full-term pregnancies (control group) and the groups of embryos with residual and enhanced growth.

Amniotic fluid BDNF levels were measured using the Quantikine Human BDNF Immunoassay (R&D Systems, Minneapolis, MN, USA) according to the manufacturer's instructions. This Elisa kit is used for cell culture supernates, tissue lysates, serum, EDTA plasma, platelet-poor EDTA plasma, heparin plasma, platelet-poor heparin plasma, urine, and human milk, so it was the most appropriate kit to be used for amniotic fluid. Even more, this ELISA kit has been used effectively for serum BDNF quantification and, given the early 2nd trimester amniotic fluid resemblance in composition with serum, it can be also used for amniotic fluid samples [28]. As the BDNF concentrations in the amniotic fluid were found significantly higher than those of serum, serial dilutions of samples were performed to demonstrate that the Elisa kit used was valid to quantify our BDNF amniotic fluid levels (linear detection). Samples required at least a 20-fold dilution into Calibrator Diluent RD6P prior to the assay. The suggested 20-fold dilution is 10 *μ*l of sample + 190 *μ*l of Calibrator Diluent RD6P. The intra-assay coefficient of variation ranged from 2.4–3.2% and the interassay coefficient of variation ranged from 4.3–7.0%, respectively, while the minimum detectable dose (MDD) of total BDNF ranged from 0.372–1.35 pg/ml.

The results were statistically evaluated by means of the SPSS statistical package using parametric and nonparametric methods, depending on indications. The Kruskal-Wallis test was used for comparison of BDNF concentrations between the three groups. Mean and standard deviations for quantitative variables are displayed in the results. We also applied linear models to examine the difference in BDNF concentrations between different degrees of growth impairment and the control group. In accordance with our study design, confounding factors, including maternal age, body mass index, duration of pregnancy, fetal sex, smoking, and multiparity, were taken into account. Lastly, the distribution of sample values was evaluated by regression analysis (Kolmogorov-Smirnov test). We set the level of significance at a *p* value of less than 0.05.

Informed consent was taken from all women who participated in the study. Furthermore, the study was approved by the Ethical Committee for Research of Aretaieio University Hospital, Athens, Greece.

## 3. Results

Eighty (80) amniotic fluid samples were measured in total. The descriptive characteristics of the mothers and fetuses are depicted in Table 1. No statistically significant differences were observed as regards maternal weight, maternal height, maternal parity, and duration of gestation among the three groups. However, maternal age, birth weight, and offspring gender were statistically different between groups. The reason for the latter findings may lie in the fact that many SGA fetuses are FGR fetuses and their birth weight is also lower, while LGA fetuses have higher than average weight. Importantly, maternal age is a well-known factor affecting fetal growth, with advanced maternal age being a risk factor for intrauterine growth restriction.

**Figure 1:**
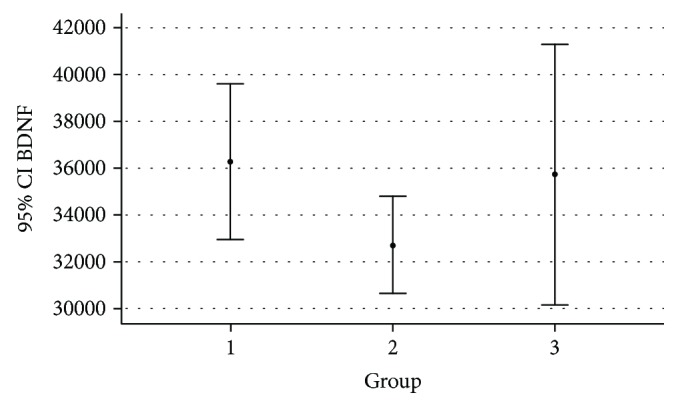
BDNF mean values (pg/ml) and 95% confidence intervals: comparison between groups (1 = SGA, 2 = AGA, 3 = LGA).

Table 2 and Figure 1 summarize the BDNF assay results showing the mean values of BDNF in the three studied groups, SGA, LGA, and control group. Notably, higher BDNF levels were detected in the amniotic fluid of both SGA and LGA fetuses as compared to normal fetuses (mean values of 36,300 pg/ml and 35,700 pg/ml vs 32,700 pg/ml for SGA and LGA fetuses vs normal fetuses, respectively).

**Figure 2:**
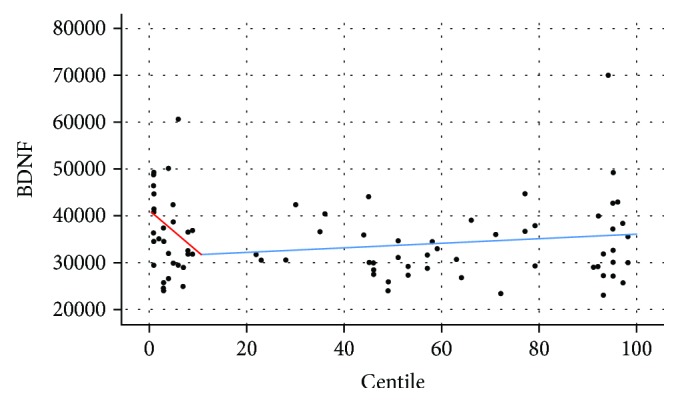
Regression analysis line graph of variables, BDNF (pg/ml) and fetal growth centile: a bimodal depiction of BDNF amniotic fluid levels is shown.

Figure 2 shows that the more severe the growth restriction, the more elevated are amniotic BDNF levels. Normally growing fetuses demonstrate a different pattern compared to growth-restricted fetuses, while fetuses with lower biometry demonstrate lower BDNF levels and those with higher biometry demonstrate higher BDNF levels (Figure 2). This tendency also applies to the fetal macrosomia group, which similarly demonstrates elevated amniotic BDNF levels (Figure 2).

In Table 3, comparison of the distribution of BDNF levels by fetal size is presented. BDNF levels are increased further in women with severe and very severe SGA fetuses. Compared with AGA fetuses, very severe SGA fetuses (below the 3^rd^ centile) display significantly higher BDNF levels. Compared with AGA fetuses, SGA fetuses below the 5^th^ centile, and even the whole group of SGA fetuses (below the 10^th^ centile), demonstrate higher BDNF. Among the group of LGA fetuses demonstrating increased levels of BDNF compared with AGA fetuses, there was no statistical significance.

## 4. Discussion

Although neurotrophins may originate from several origins, like maternal, placental, or fetal compartments, there is evidence that BDNF in second trimester amniotic fluid is mainly of fetal origin [29]. Consequently, second trimester amniotic fluid evaluation reliably reflects the fetal condition and its central nervous system underneath chemo-biological mechanisms in cases of impaired endometrial growth or macrosomia. Research findings further suggest that circulating amniotic fluid neurotrophins can affect fetal neurodevelopment during pregnancy [30].

To date, while BDNF measurements have been carried out in neonates and adult humans, as well as in animal fetal studies, correlating these with several aspects of neural growth and function, there has been no corresponding human fetal research regarding associations with fetal growth and adaptation to a hostile intrauterine environment. Furthermore, there is also a lack of data concerning the role of neurotrophic factors in macrosomia. Our aim was therefore to measure the amniotic fluid levels of the neurotrophic factor BDNF and to investigate their association with fetal growth.

BDNF and its TrkB receptor are widely expressed in both the developing and the adult mammalian brain, with BDNF/TrkB-stimulated intracellular signaling being critical for neuronal activity as well as for neuronal plasticity, protection, metabolism, and survival [31]. Also crucially, BDNF-positive neurons participate in the early development of the frontal lobe of the human fetal cerebrum. During the fourth month of gestation, at which stage our amniocentesis was performed, BDNF-positive neurons grow larger in size and BDNF-positive expression is enhanced [32]. It has further been proposed that maternal BDNF, by reaching the fetal brain via the utero-placental barrier, possibly thereby supports the development of the fetal central nervous system [33].

Fetal growth restriction (FGR) describes the condition in which a fetus is unable to reach its genetically predetermined growth potential. This may be due to anatomical or functional diseases in the fetal-placental unit, whereby the fetus adapts its circulation to redistribute oxygen, fetal blood flow, and nutrient supply to the vital organs, i.e., the myocardium, brain, and adrenal glands, this phenomenon known as the brain-sparing effect. When this condition persists, it brings about FGR. Given that the brain-sparing effect sometimes occurs in full-term FGR infants, circulating neurotrophin levels should be similar between late nonsevere FGR infants and AGA infants. Previous studies have in fact demonstrated that both groups display similar levels of circulating BDNF, a finding possibly attributable to the activation of the brain-sparing effect [34]. Nevertheless, there is still uncertainty as regards the triggering of this response in early or very severe FGR fetuses and macrosomic fetuses.

The neurotrophin family apart from BDNF and NGF is composed of two more structurally related molecules: neurotrophin-3 (NT-3) and neurotrophin-4 (NT-4). Because they exert neuroprotection, neurotrophins play a crucial role in pre- and postnatal brain development. The BDNF neuroprotective effect involves a number of pathophysiologic aspects that include apoptosis, inflammation, intracellular metabolism, and regeneration procedures. In particular, there is strong evidence showing the beneficial impact of BDNF on the survival of neurons via the antiapoptotic effect. The means by which BDNF reduces neuron apoptosis is by enhancing the expression of the Bcl-2 antiapoptosis protein while limiting intracellular calcium overload [35].

Growth-restricted fetuses may exhibit fetal compromise, with hypoxia being the main underlying pathophysiological mechanism. It has been demonstrated in vitro that in the setting of hypoxia-induced inflammation, BDNF stimulates microglial proliferation and phagocytic activity while elevating the number of phagocytotic microglia and activated microglia, which themselves secrete BDNF [36]. BDNF can suppress TNF-*α* and its mRNA expression, this exacerbating ischemia-induced injury, while it increases IL-10 and its mRNA expression, which play an anti-inflammatory neuroprotective role [37]. The putative mechanism behind damage to the developing brain is a neuroinflammatory response in the fetal central nervous system resulting from fetal infection and/or systemic inflammation [38]. What is more, BDNF participates in the parallel activation of anti-inflammatory mechanisms, which are thought to provide negative feedback loops as well as to induce neuroprotective effects and possibly also repair mechanisms in the developing brain [39].

Glucose depletion and ischemia, by bringing about primary energy failure, trigger a cascade of biochemical events, leading to cell dysfunction. Meanwhile, a resultant reperfusion injury often impairs brain metabolism aggravating the damage caused by oxidative stress, with the main mediator of oxidative stress damage being nitric oxide (NO) [40, 41]. BDNF resists NO-mediated glutamate metabolic cytotoxicity depending on its concentration [42], the latter comprising a possible neuroprotective role of BDNF in cases of FGR fetuses who present an activated brain-sparing effect, as well as macrosomic fetuses, especially in cases of maternal diabetes. As neurogenesis involves cell proliferation, migration, and differentiation, the augmentation of BDNF around the injured region is critical in facilitating regeneration among central and peripheral neurons [43, 44]. As proinflammatory cytokines, such as TNF-*α*, IL-1, IL-6, and IL-11, cause serious damage to the capillary endothelium and the alveolar epithelium [45], BDNF may also protect the fetal respiratory system.

Both in vitro and in vivo animal studies have revealed BDNF to be involved in embryo implantation, placental development, and fetal growth through its stimulation of blastocyst outgrowth [46, 47] and trophoblast cell growth and survival [48], as well as being necessary for further placental development [49]. Studies on human FGR placentas have shown that expressions of both BDNF and its TrkB receptor mRNA are upregulated [50].

According to our findings, it seems that an adaptive mechanism accelerates fetal brain development and maturation, a process that is induced by growth restriction chronic hypoxia, this mechanism, expressed by increased BDNF levels, becoming even more enhanced as the growth restriction gets more critical. Interestingly, the above findings are in accord with neonatal studies demonstrating that SGA infants have significantly higher BDNF levels than AGA infants [51].

Severely growth-restricted fetuses display a pattern of several growth factor disturbances, including PLGF deficiency [52, 53]. Moreover, severely low concentrations of PLGF have been associated with impaired angiogenesis, placentation, and placental development, resulting in complications, notably fetal growth restriction [53, 54]. Meanwhile, there is partial modulation by PLGF of vascular endothelial growth factor (VEGF) activity, the latter factor representing the most potent mediator of angiogenesis [54]. BDNF, a known promoter of endothelial cell survival, induces neoangiogenesis in ischemic tissues, thus complimenting the development of the growth-restricted fetus [55]. BDNF and NGF have, moreover, been implicated in the modulation of angiogenesis [56].

According to our findings, fetal macrosomia also correlates with elevated BDNF levels, providing a mirror image of BDNF amniotic fluid levels as the fetal growth centile increases, the latter possibly reflecting the advanced fetal and placental mass. Diabetes is a major cause of fetal macrosomia and, by extension, even perinatal morbidity. In addition, intrauterine exposure to a diabetic environment during pregnancy can impact the child long term, since it is associated in the offspring with subclinical vascular inflammation and endothelial dysfunction which are linked to the development of cardiovascular disease later in life [57]. BDNF, as an anti-inflammatory mediator whose levels are increased in macrosomic and diabetic pregnancies, may partially reverse these consequences.

## 5. Conclusions

Our study is the first to confirm the presence of the neurotrophic factor BDNF in the amniotic fluid of early midtrimester human pregnancies, with significantly higher BDNF levels being observed in the amniotic fluid of severely growth-restricted fetuses compared to normal fetuses. This concerns a compensatory and adaptive mechanism, induced by growth restriction, which accelerates fetal brain development and maturation. Normally growing and macrosomic fetuses demonstrate a different BDNF pattern from FGR fetuses, leading to a bimodal depiction of BDNF amniotic fluid levels as the fetal growth centile changes.

Through BDNF-stimulated intracellular signaling, BDNF, a neurotrophin widely expressed in the developing fetal brain, plays a vital role in supporting neuronal formation, protection, and metabolism, while it additionally has a major role in placental development and fetal growth. From the clinical perspective, BDNF has been associated with the pathophysiology of a number of pregnancy complications, such as low birth weight and growth restriction. However, it also demonstrates promise as a potential prognostic factor involved in the mechanisms underlying fetal growth restriction and macrosomia, which often give rise to maternal, fetal, and neonatal adverse outcomes. Further studies validating our results and those of previous studies will provide enhanced insight into the processes underlying fetal growth.

## Figures and Tables

**Table 1:** Comparative characteristics between groups SGA, LGA, and Control (mean values ± SD).

Variable	SGA group *N* = 31	LGA group *N* = 18	Control group *N* = 31	*P* value
Maternal age	37 ± 3.4	33.5 ± 5	34.5 ± 3.4	0.01
Maternal weight	69.3 ± 14.7	59.9 ± 7.9	65.1 ± 11.1	0.15
Maternal height	166.8 ± 5	164.7 ± 7.1	167 ± 5.6	0.60
Maternal parity	0.9 ± 0.9	0.7 ± 0.7	0.7 ± 1	0.24
Fetal sex	Mostly XX	Mostly XY	Mostly XY	0.02
Birth week	35.5 ± 9.7	37.8 ± 1.1	38.5 ± 0.8	0.11
Birth weight (in grams)	2363.9 ± 746.3	3870.6 ± 335.2	3332.3 ± 285.1	0.01

**Table 2:** Amniotic fluid BDNF mean values in the three studied groups: SGA, LGA, and Control: both SGA and macrosomic fetuses are characterized by notably higher amniotic fluid levels of BDNF compared to normal-growth fetuses.

Group	*N*	BDNF (mean value ± SD)	*P* value
SGA	31	36,300 ± 9000 pg/ml	0.09
LGA	18	35,700 ± 11,200 pg/ml	0.22
Control	31	32,700 ± 5700 pg/ml	

**Table 3:** Distribution of BDNF (pg/ml) by fetal size group, including the extremes of distribution (divided into three subgroups for each 0^th^–10^th^ and 90^th^–100^th^ centile): significantly higher BDNF levels are observed in the amniotic fluid of severely growth-restricted fetuses (below 3^rd^ centile).

Fetus	*N* of cases	Mean	*P* value
AGA	31	32,700	
SGA < 10^th^ centile	30	36,300	0.09
SGA < 5^th^ centile	18	36,900	0.09
SGA < 3^rd^ centile	10	40,800	0.01
LGA > 90^th^ centile	18	35,700	0.22
LGA > 95^th^ centile	5	34,600	0.65
LGA > 97^th^ centile	2	35,700	0.53

## Data Availability

The data used to support the findings of this study are available from the corresponding author upon request.
